# Comparison of the transcriptome and metabolome of wheat (*Triticum aestivum* L.) proteins content during grain formation provides insight

**DOI:** 10.3389/fpls.2023.1309678

**Published:** 2024-01-18

**Authors:** Jia Shi, Lihong Wang, Zhong Wang, Jianfeng Li, Hongzhi Zhang, Xin Gao, Chunsheng Wang, Jianqiang Xia, Zhun Zhao, Zhenlong Wang, Zhenyu Yang, Zihan Xu, Yueqiang Zhang, Zheru Fan

**Affiliations:** Institute of Nuclear and Biological Technologies, Xinjiang Academy of Agricultural Sciences/Xinjiang Key Laboratory of Crop Biotechnology/Crop Chemical Regulation Engineering Technology Research Center in Xinjiang, Urumqi, China

**Keywords:** wheat, transcriptome, metabolome, glutenin content, WGCNA

## Abstract

**Introduction:**

Wheat is a food crop with a large global cultivation area, and the content and quality of wheat glutenin accumulation are important indicators of the quality of wheat flour.

**Methods:**

To elucidate the gene expression regulation and metabolic characteristics related to the gluten content during wheat grain formation, transcriptomic and metabolomic analyses were performed for the high gluten content of the Xinchun 26 cultivar and the low proteins content of the Xinchun 34 cultivar at three periods (7 d, 14 d and 21 d) after flowering.

**Results:**

Transcriptomic analysis revealed that 5573 unique differentially expressed genes (DEGs) were divided into two categories according to their expression patterns during the three periods. The metabolites detected were mainly divided into 12 classes. Lipid and lipid-like molecule levels and phenylpropanoid and polyketide levels were the highest, and the difference analysis revealed a total of 10 differentially regulated metabolites (DRMs) over the three periods. Joint analysis revealed that the DEGs and DRMs were significantly enriched in starch and sucrose metabolism; the citrate cycle; carbon fixation in photosynthetic organisms; and alanine, aspartate and glutamate metabolism pathways. The genes and contents of the sucrose and gluten synthesis pathways were analysed, and the correlation between gluten content and its related genes was calculated. Based on weighted correlation network analysis (WGCNA), by constructing a coexpression network, a total of 5 specific modules and 8 candidate genes that were strongly correlated with the three developmental stages of wheat grain were identified.

**Discussion:**

This study provides new insights into the role of glutenin content in wheat grain formation and reveals potential regulatory pathways and candidate genes involved in this developmental process.

## 1 Introduction

Wheat (*Triticum aestivum* L.) is the largest crop in the world, providing approximately 20% of the food available for humankind and one of the most important food crops (Chawade et al., 2018). With the improvements in living standards, the quality of wheat has received increasing attention (Chawade et al., 2018). Wheat grain protein is divided into albumin, globulin, gliadin and gluten according to solubility; gliadin and gluten are the main storage proteins and are the main components of gluten (Zheng et al., 2018; Hackenberg et al., 2019). Because of the presence of gliadin and gluten components, wheat flour can be kneaded with water to form a dough, which can be fermented, steamed or baked to obtain a variety of foods (Zheng et al., 2018; Hackenberg et al., 2019). The content and quality of gluten are important indicators for determining the quality of wheat flour, determining the process performance of dough and determining the quality of steamed and baked goods (Zheng et al., 2018; Hackenberg et al., 2019). Gluten imparts characteristics of water retention, cohesion, viscoelasticity, etc., that play a decisive role in the rheological properties and baking quality of dough (Zheng et al., 2018; Hackenberg et al., 2019). Therefore, exploring candidate genes for the study of wheat gluten content, analysing the underlying molecular mechanism, and improving the quality of wheat cultivars through breeding pathways are important tasks for modern wheat breeding.

Transcriptomics is a discipline that studies gene expression and transcriptional regulation in cells as a whole, and transcriptome analysis is necessary for exploring genome function and differential expression and plays an important role in studying plant growth and development (Chen et al., 2014; Pankievicz et al., 2016; Stelpflug et al., 2016; Hsu and Tung, 2017; Ji et al., 2022). Metabolomics refers to the inheritance and development of genomics, transcriptomics and proteomics and can directly reflect the biochemical pathways and potential molecular mechanisms in organisms by elucidating the metabolites downstream of the genome as a whole and subsequently revealing the relevant metabolic pathways and metabolic networks (Li et al., 2022; Prakash et al., 2023). In recent years, transcriptomic and metabolomic-based techniques have provided powerful tools and methods for revealing molecular characteristics and identifying candidate genes related to plant growth and development and fruit quality (Jiang et al., 2022; Wan et al., 2022; Zhang and Fernie, 2023). Coexpression network analysis is a systems biology method in which gene coexpression networks are constructed by analysing the correlation of gene expression to discover functionally relevant gene modules (Ma et al., 2021). Transcriptome and metabolome techniques have been used to detect differences in fruit flavour and carotenoid content in the early ripening (MG) and postripening (TR) stages of mango fruits (Peng et al., 2022). Transcriptome and metabolome data were used to study the accumulation of metabolites and transcriptional changes in the late-maturing cultivar Kate Mango at different stages of fruit development, and a regulatory network related to mango fruit ripening was constructed (Wu et al., 2022). By analysing clusters of metabolites and genes with the same tendencies to change in expression in cashews, 17 genes involved in phosphatidylinositol (PI) synthesis were found, and the transcription factor *WRKY11*, which can potentially regulate PI synthesis, was also identified (Zhao et al., 2022). Through poplar transcriptomics and metabolomics, the effects of miR156 on other microRNAs and their targets associated with anthocyanin biosynthesis were revealed (Wang et al., 2020).

The gluten content during the process of wheat grain formation is an important index for quality evaluation, and studying the gluten content is highly important for improving the quality of wheat (Zheng et al., 2018; Hackenberg et al., 2019). However, the gluten content during wheat grain formation involves complex polygenetic mechanisms, multisignalling pathways and metabolic processes. Therefore, in this study, the high-gluten-content Xinchun 26 cultivar and the low-gluten-content Xinchun 34 cultivar were selected for application of transcriptomic and metabolomic methods to conduct a cluster analysis of differentially expressed genes (DEGs) and differentially regulated metabolites (DRMs), KEGG enrichment analysis, and transcription factor (TF) expression analysis and to determine the key genes related to gluten content in wheat grain formation through coexpression analysis and qRT−PCR. This study provides new insights into the gluten content during wheat grain formation and reveals potential regulatory pathways and candidate genes involved in this developmental process.

## 2 Materials and methods

### 2.1 Plant material

The high gluten spring wheat cultivar Xinchun 26 and the low gluten spring wheat cultivar Xinchun 34 were chosen for the study. The above two cultivars were sown according to the designated community area of 4.8 m^2^ in the military household experimental base of Changji city, Xinjiang, and the management method was the same as that used for conventional fields. Fertilization and watering were applied at the same time to ensure that the growth environment of the two cultivars was the same. Mid-spike grains exhibiting consistent growth were collected at 7 d, 14 d, and 21 d after flowering, and 14 replicates were collected for each variety (3 for RNA-seq sequencing, 5 for metabolome sequencing, 3 for physiological index determination, and 3 for qRT−PCR). Immediately after collection, the samples were flash frozen with liquid nitrogen, brought back to the laboratory and stored in a -80°C freezer.

### 2.2 RNA-seq sequencing and analysis

After DNase I (Illumina, USA) digestion of the sample total RNA, the mRNA was purified from 1 μg of total RNA using oligo (dT) magnetic beads, followed by mRNA fragmentation in ABclonal First Strand Synthesis Reaction Buffer. Subsequently, the first strand of cDNA was synthesized with random primers and reverse transcriptase (RNase H) using fragmented mRNA as a template, and the second strand of cDNA was subsequently synthesized with dNTPs, RNAseH, DNA polymerase I and buffer and ligated to perform PCR amplification. The PCR products were purified, and the library quality was evaluated using an Agilent Bioanalyzer 4150 (Kusser et al., 2006). The constructed library was sequenced on the Illumina HiSeq 2500 sequencing platform. Sequencing was performed by Nanjing Jisi Huiyuan Biotechnology Co., Ltd. (Nanjing, China). After the original sequence was obtained, Fastp software (version 0.23.4) was used to remove the barcode sequence and filter out the N sequences with low masses and ratios greater than 5%, etc., to obtain clean reads that could be used for subsequent analysis (Chen et al., 2018). HISAT2 was used to align the clean reads with the wheat reference genome (https://urgi.versailles.inra.fr/download/iwgsc/IWGSC_RefSeq_Assemblies/v2.1/, version iwgsc_refseqv2.1) (Pertea et al., 2016). The number of transcripts per thousand bases per million mapped fragments (FPKM) was used for the characterization of expression. The read counts (raw counts) of the genes were calculated, and the p values and fold changes were calculated with DESeq2 software. A P value ≤ 0.05 and |log2fold change|>1 were used as the screening criteria for identifying DEGs (Liu et al., 2021). The DEGs were annotated based on the KEGG database (http://www.genome.jp/kegg/) (Kanehisa and Goto, 2000).

### 2.3 Metabolite extraction

One hundred milligrams of the sample was measured, and 800 μL of the extraction solution (methanol–acetonitrile–water volume ratio = 2:2:1, internal standard concentration = 20 mg/L) was added to the internal standard and added to each sample. Two small steel balls were added, and the samples were placed into a tissue grinder for grinding (at 50 Hz for 5 min; special samples that are difficult to break can be appropriately extended). After ultrasonication in a 4°C water bath for 10 min, the samples were allowed to rest at -20°C for 1 h. The samples were subsequently centrifuged at 4°C at 25,000 rpm for 15 min. After centrifugation, 600 μL of the supernatant was added to a 96-well plate. Using a 96-well filter plate for filtration, 200 µl of 70% methanol was first added to rinse the filter plate, and then 500 µl of supernatant was added for filtration. The filtered samples were collected, and each sample was transferred to a 96-well plate with 100 μL of supernatant. The plates were divided into positive and negative ions and were spared for a total of 3 plates (Jones and Kinghorn, 2012).

### 2.4 UPLC−MS analysis

In this experiment, a Waters UPLC I-Class Plus (Waters, USA) tandem Q Autonomous high-resolution mass spectrometer (Thermo Fisher Scientific, USA) was used for the separation and detection of metabolites. The column used was a Hypersil GOLD aQ Dim column (1.9 μm 2.1*100 mm, Thermo Fisher Scientific, USA). The mobile phases were 0.1% formic acid in water (liquid A) and acetonitrile (liquid B) containing 0.1% formic acid. The flow rate was 0.3 mL/min, the column temperature was 40°C, and the injection volume was 5 μL. A Q Autonomous mass spectrometer (Thermo Fisher Scientific, USA) was used for primary and secondary mass spectrometry data acquisition. The mass spectrometry scanning mass-core ratio range was 125~1500 positive ions, 100~1500 negative ions, 70,000 first-order resolution, 1e6 AGC, and 100 ms injection time (IT, injection time). The MS data were imported into Compound Discoverer 3.2 (Thermo Fisher Scientific, USA) software, combined with the mzCloud database and the ChemSpider online database for MS data analysis, and a data matrix containing the metabolite peak area and identification results was obtained (Jones and Kinghorn, 2012; Wen et al., 2017; Huang et al., 2019; Yu et al., 2021).

### 2.5 Metabolomic analysis

Based on the metabolite content data matrix, principal component analysis (PCA) was performed on each sample using R. The first principal component was first modelled and analysed by OPLS-DA, and the quality of the model was tested by 7-fold cross-validation. The validity of the model was judged by the R2Y (interpretability of the model to the categorical variable Y) and Q2 (predictability of the model) obtained by cross-validation. Finally, by permutation test, the order of the categorical variable Y was randomly adjusted several times to obtain different random Q2 values, and further tests of the effectiveness of the model were performed. Using the Human Metabolome Database (HMDB) and KEGG database, the classification of metabolites and the functional annotation of the pathway were carried out, and the main biochemical metabolic pathways and signal transduction pathways associated with the metabolites were determined (Kanehisa and Goto, 2000). Partial least squares regression was used to establish a model of the relationship between metabolite expression and sample class to model and predict sample class (Barker and Rayens, 2003; Westerhuis et al., 2008). The fold change in the expression of each metabolite in each comparison group was calculated. Student’s t test was used to test the significance of the expression of each metabolite in each comparison group, and a fold change ≥1.2 or ≤0.83 and a q value < 0.05 were used as the standards for screening for differentially abundant metabolites (Dunn et al., 2011).

### 2.6 WGCNA

To ensure the distribution of scale-free networks, the weighting coefficient β should meet the correlation coefficient close to 0.8 and have a certain degree of gene connectivity. In this study, β=7 was selected as the weighting coefficient. The automatic network building function of blockwise modules was used to construct the network, and multiple valid modules were obtained. The number of genes contained in each module was different. MinModuleSize = 30 and Merge Cut Height = 0.25 were used as the standards, and modules with a combined similarity of 0.75 were obtained. The correlation coefficients between the module’s characteristic vector ME (module eigengene) and different durations of hormone content and treatment were calculated. R>0.80 and P<0.05 were used as criteria for screening the specificity modules. Cytoscape (version 3.10.0) software was used for visualization of coexpression networks (Shannon et al., 2003).

### 2.7 qRT−PCR

Total RNA was extracted using an EZNA. Plant RNA Kit (Omega Bio-Tek, Doraville, GA, USA). The concentration of each RNA sample was determined using a NanoDrop 2000 spectrophotometer (Thermo Fisher Scientific, Waltham, MA, USA), followed by the use of 1 μg of isolated RNA to obtain first-strand cDNA via a PrimeScript reverse transcription RT kit with gDNA™ erasure (Takara Bio, Inc., Shiga, Japan). qRT−PCR analysis was performed using Roche LC480 equipment (Roche Diagnostics GmbH, Mannheim, Germany) and SYBR Green (Takara Bio, Inc.). Using a two-step PCR amplification procedure, predenaturation was carried out at 95°C for 30 sec, followed by 40 cycles of denaturation at 95°C for 5 sec and annealing at 60°C for 34 sec. The relative expression levels of the target genes were calculated using geNorm software, with the reference gene Actin and three biological replicates for each gene. All primers used in this study are shown in **Supplementary Table S1**.

## 3 Results

### 3.1 Determination of Xinchun 26 and Xinchun 34 protein content

**Figure 1:**
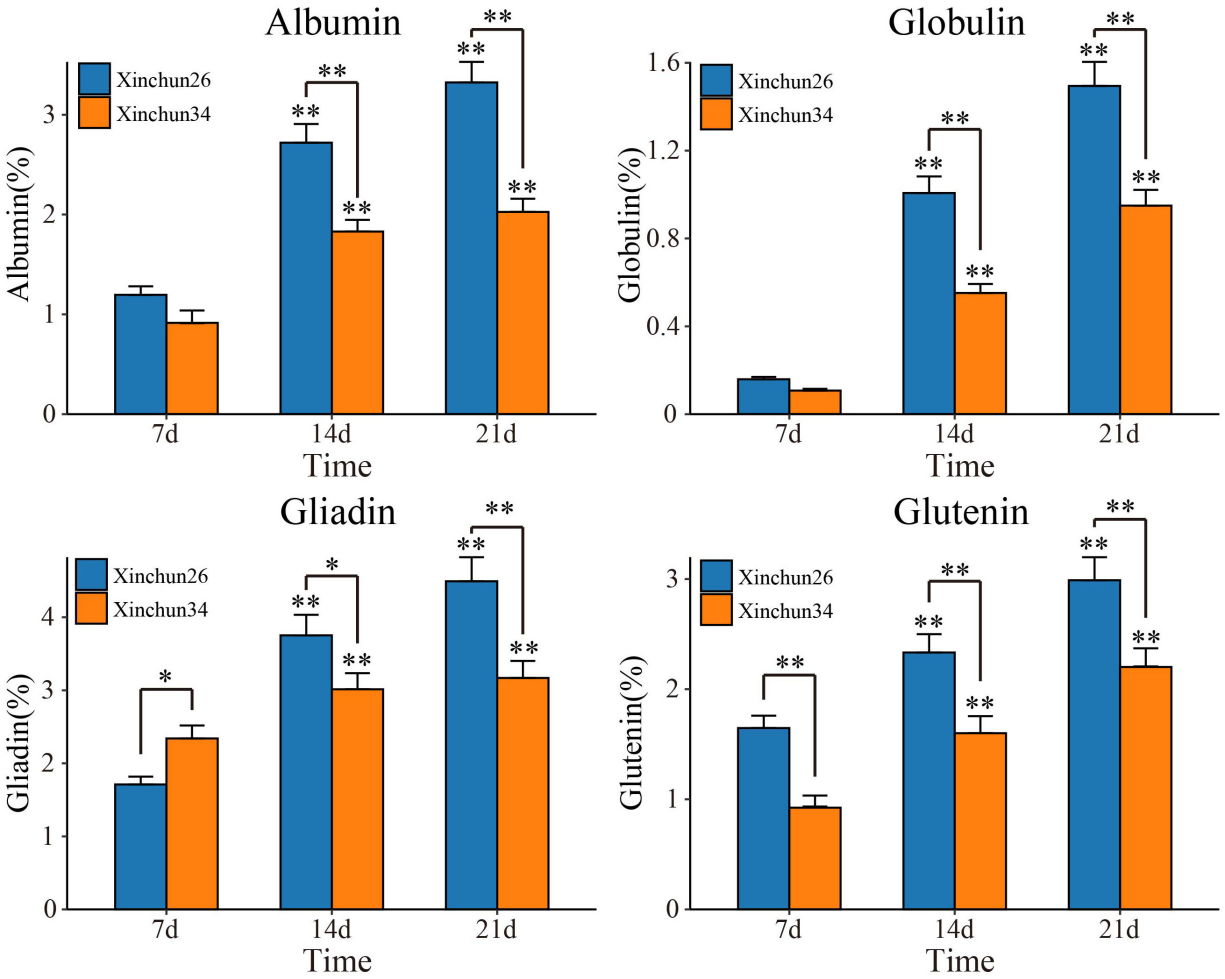
Albumin, globulin, gliadin and glutenin contents of Xinchun 26 and Xinchun 34 seeds 7 d, 14 d and 21 d after flowering. The results are presented as the means ± SDs (n = 3, **P < 0.01).

The content and quality of gluten are important indicators for determining the quality of wheat flour and determine the process performance of the dough and the quality of steamed and baked goods (Zheng et al., 2018; Hackenberg et al., 2019). To do this, we first determined the levels of four proteins (albumin, globulin, gliadin and glutenin) in Xinchun 26 and Xinchun 34 seeds 7 d, 14 d and 21 d after flowering (**Figure 1**). Compared with those at 7 d after flowering, the expression of the four proteins at 14 d and 21 d increased significantly in both materials. The serum ALB concentration significantly differed among the three treatment groups, and the globulin concentration significantly differed between the two treatment groups at 21 d. Gliadin and glutamine levels were significantly greater in Xinchun 26 than in Xinchun 34 at 7 d, 14 d and 21 d. These results showed that the gluten content in Xinchun 26 was significantly greater than that in Xinchun 34. To further explore key genes and key metabolites related to gluten content during wheat grain formation, RNA-seq and metabolome sequencing were performed on Xinchun 26 and Xinchun 34 grains at 7 d, 14 d and 21 d after flowering.

### 3.2 RNA-seq analysis

A total of 18 RNA-seq samples from 2 materials and 3 periods produced a total of 130.37 Gb of data, and the amount of clean data from each sample reached 5.45 Gb or more. The Q30 percentage was 89.52%, the percentage of sequences that were shared with the reference genome was between 86.08% and 94.31%, and the average alignment rate was 89.62% (**Supplementary Table S2**). The correlation between the same biological replicates was good, the correlation coefficient range was 0.84~1.00, the PCA and correlation analysis results were consistent, and the replicates were clustered together (**Supplementary Figure S1**). Ten genes were randomly selected for 3 independent replicates of qRT−PCR analysis, and the transcriptome data were significantly correlated with the qRT−PCR data (R^2 =^ 0.9207; **Supplementary Figure S2**). The results showed that the test sampling was reasonable and that the RNA-seq data quality was reliable.

### 3.3 RNA-seq differential analysis

**Figure 2:**
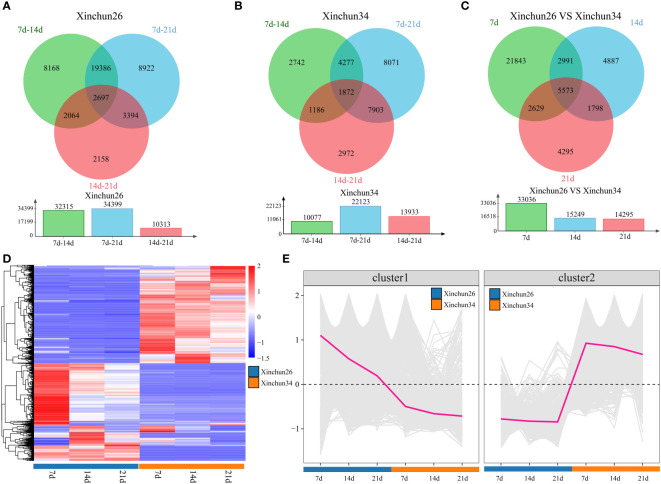
**(A)** There were Xinchun (26) DEGs at 7 d, 14 d and 21 d after flowering, **(B)** There were Xinchun (34) DEGs and corresponding quantities at 7 d, 14 d and 21 d after flowering, **(C)** The Venn diagrams and quantities of DEGs in Xinchun (26) and Xinchun (34) flowers after 7, 14 and 21 d, **(D)** shows an expression heatmap of DEGs between 7 d, 14 d and 21 d after Xinchun 26 and Xinchun 34 treatment, **(E)** Line chart of the expression trends of DEGs among the 7 d, 14 d and 21 d periods after Xinchun 26 and Xinchun 34 flowered.

Differential analysis was performed over 3 developmental periods for Xinchun 26, which revealed 32,315 DEGs between 7 d and 14 d, 34,399 DEGs between 7 d and 21 d, and 10,313 DEGs between 14 d and 21 d, for a total of 2,697 DEGs over three periods (**Figure 2A**). In Xinchun 34, there were 10,077 DEGs between 7 d and 14 d, 22,123 DEGs between 7 d and 21 d, and 13,933 DEGs between 14 d and 21 d, for a total of 1,872 DEGs (**Figure 2B**) over the three periods. Among the two materials, 7 d had 33036 DEGs, 14 d had 15249 DEGs, 21 d had 14925 DEGs, and 5573 DEGs were detected over the three periods (**Figure 2C**). A total of 5573 DEGs in the three periods were divided into two categories according to their expression patterns; the expression of Cluster 1 in Xinchun 26 was greater than that in Xinchun 34, and the expression gradually decreased with the development of grains (**Figures 2D**, **E**). Similarly, the expression of Cluster2 in Xinchun 34 was greater than that in Xinchun 26, but the expression did not change with grain development (**Figures 2D**, **E**).

### 3.4 Metabolomic analysis

**Figure 3:**
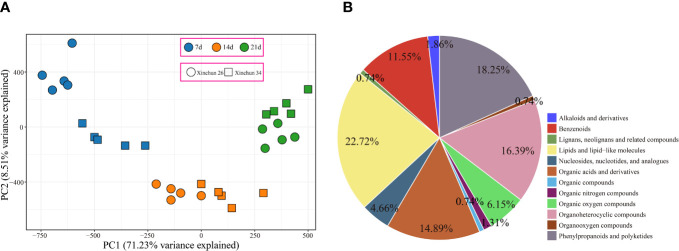
**(A)** Metabolome PCA. **(B)** Metabolome classification pie chart.

UPLC−MS identified a total of 863 metabolites, and PCA revealed that the first principal component could explain 71.23% of the total variance, the second principal component could explain 8.51% of the total variance, and the first principal component could distinguish different materials and periods (**Figure 3A**). To understand the classification and functional characteristics of the different metabolites, we classified and annotated the identified metabolites, which were divided into 12 main categories. The contents of lipids and lipid-like molecules accounted for 22.72%, the contents of phenylpropanoids and polyketides accounted for 18.25%, the contents of organoheterocyclic compounds accounted for 16.39%, and the contents of organic acids and derivatives accounted for 14.89%. Benzenoids accounted for 11.55%; organic oxygen compounds, 6.15%; nucleosides, nucleotides, and analogues, 4.66%; alkaloids and derivatives, 1.86%; and organic nitrogen compounds, 1.31%. The percentages of organooxygen compounds, lignans, neolignans, related compounds and organic compounds were 0.74% (**Figure 3B**).

### 3.5 Metabolomic difference analysis

**Figure 4:**
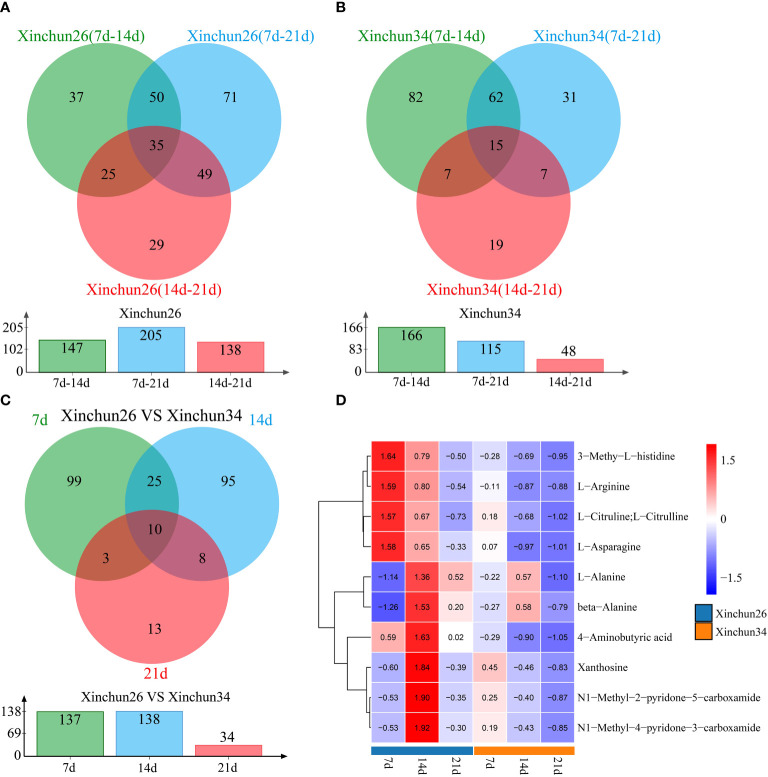
**(A)** Xinchun 26, 7, 14 and 21 d after flowering, a Venn diagram and quantity of DRMs were generated, **(B)** Xinchun 34, 7, 14 and 21 d after flowering, a Venn diagram and number of DRMs were generated, **(C)** The Venn diagrams and quantities of the Xinchun 26 and Xinchun 34 flowers after 7, 14 and 21 d of treatment, **(D)**. Heatmap of DRM levels 7, 14 and 21 d after Xinchun 26 and Xinchun 34 flowering.

Differences were observed over 3 developmental periods in Xinchun 26, with 147 DRMs occurring between the 7 d mark and 14 d mark, 205 DRMs occurring between the 7 d mark and 21 d mark, 138 DRMs occurring between the 14 d mark and 21 d mark, and a total of 35 DRMs occurring over the three periods (**Figure 4A**). In Xinchun 34, there were 166 DRMs between 7 d and 14 d, 115 DRMs between 7 d and 21 d, 48 DRMs between 14 d and 21 d, and 17 DRMs in the three periods (**Figure 4B**). Among the two materials, 7 d had 137 DRMs, 14 d had 138 DRMs, 21 d had 34 DRMs, and 10 DRMs occurred during the three periods (**Figure 4C**). The four DRMs (3-methyl-L-histidine, L-arginine, L-citrulline, L-citrulline and L-asparagine) had the highest 7d content in Xinchun26, which decreased with development (**Figure 4D**). The six DRMs (L-alanine, beta-alanine, 4-aminobutyric acid, xanthosine, N1-methyl-2-pyridone-5-carboxamide and N1-methyl-4-pyridone-3-carboxamide) had the highest 14d content in Xinchun 26 (**Figure 4D**).

### 3.6 RNA-seq and metabolome combined analysis

**Figure 5:**
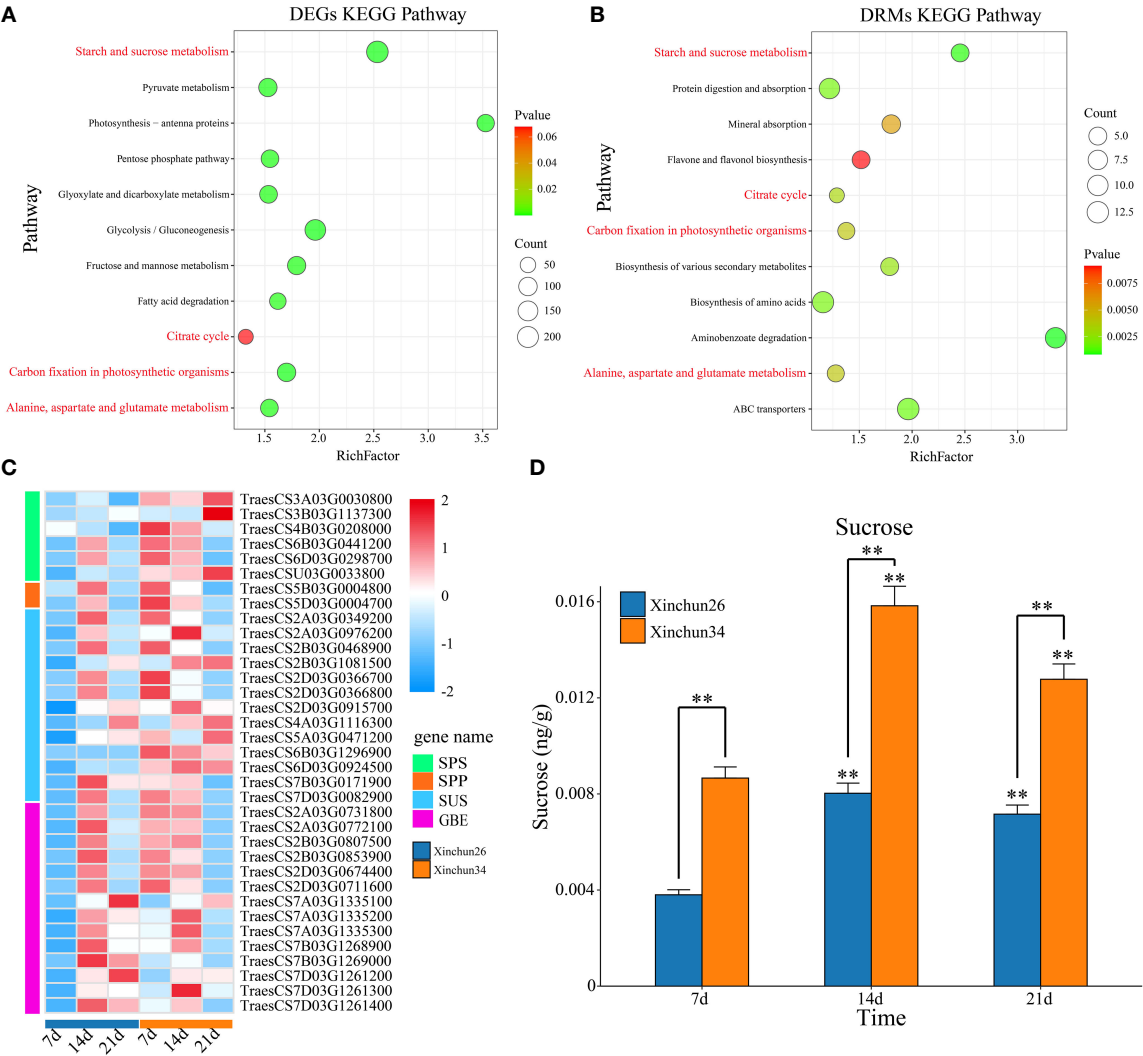
**(A)** Bubble map of DEG KEGG enrichment between materials. **(B)** Bubble map of the DRM KEGG enrichment between materials. **(C)** Gene expression calorimetry of the sucrose synthesis pathway, **(D)** The results are presented as the means ± SDs (n = 3, **P < 0.01).

KEGG enrichment analysis was performed on the DRMs and DEGs, and the DEGs were enriched mainly in starch and sucrose metabolism; photosynthesis-antenna proteins; glycolysis/gluconeogenesis; carbon fixation in photosynthetic organisms; pyruvate metabolism; fructose and mannose metabolism; the pentose phosphate pathway; alanine, aspartate and glutamate metabolism; glyoxylate and dicarboxylate metabolism; fatty acid degradation; and the citrate cycle (**Figure 5A**). The DRMs were mainly enriched in aminobenzoate degradation; starch and sucrose metabolism; ABC transporters; protein digestion and absorption; biosynthesis of amino acids; biosynthesis of various secondary metabolites; the citrate cycle; carbon fixation in photosynthetic organisms; alanine, aspartate and glutamate metabolism; mineral absorption; and flavone and flavonol biosynthesis (**Figure 5B**). The common enrichment pathways for DRMs and DEGs were involved in starch and sucrose metabolism; the citrate cycle; carbon fixation in photosynthetic organisms; and alanine, aspartate and glutamate metabolism (**Figures 5A, B**). The sucrose synthesis pathway genes and sucrose content were analysed, and the sucrose synthesis pathway genes were sucrose phosphate synthase (SPS), sucrose-phosphate phosphatase (SPP), sucrose synthase (SUS) and 1,4-alpha-glucan branching enzyme (GEB) (**Figure 5C**, **Supplementary Table S3**). The sucrose content in Xinchun 34 was significantly greater than that in Xinchun 26, and the sucrose content in both materials at 14 d and 21 d was significantly greater than that in the seeds of rose flowers at 7 d (**Figure 5D**).

### 3.7 Gluten-related gene expression analysis

**Figure 6:**
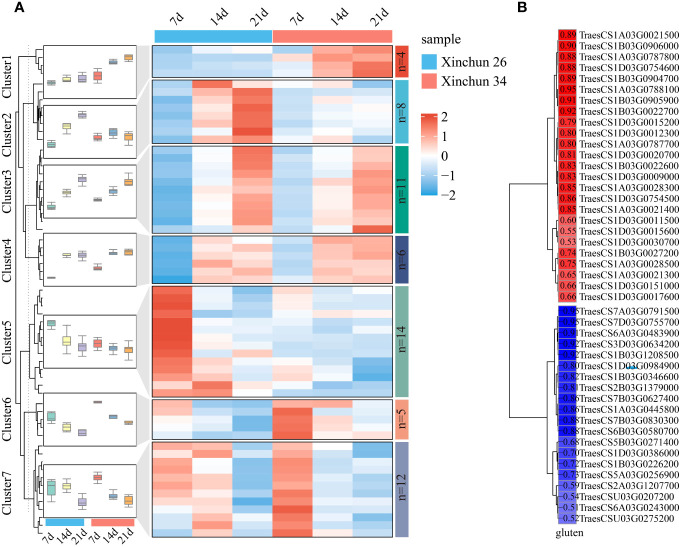
**(A)** A heatmap of the expression patterns of gluten-related genes in wheat was constructed, and the box plot represents the overall expression trend of genes in each cluster. **(B)** For the correlation between gluten-related gene expression and gluten content, red represents a positive correlation, blue represents a negative correlation, and colour depth represents the size of the image relationship number.

Among the wheat storage proteins, gluten plays a key role in the processing quality of wheat. For this purpose, glutenin-related genes were analysed among the DEGs, and a total of 59 genes were identified. The expression patterns of glutenin-related genes were visualized using a heatmap, which was divided into 7 main clusters (**Figure 6A**, **Supplementary Table S3**). Cluster 1 included 4 genes; the expression of 4 genes in Xinchun 34 was greater than that in Xinchun 26, and the expression level gradually increased with the development of grains. Cluster 2 included 8 genes, the expression level of Cluster 2 gradually increased with the development of grains in Xinchun 26, and the expression trend in Xinchun 34 remained basically unchanged. Cluster 3 included 11 genes whose expression levels increased with grain development, and the expression patterns of the two materials were basically the same. Cluster 4 included 6 genes, and with increasing grain development, the expression patterns of the two materials were basically the same, while the expression levels at 14 d and 21 d were basically unchanged. Cluster 5 included 14 genes, the expression level of Cluster 5 gradually decreased with the development of grains, and the expression level in Xinchun 26 was greater than that in Xinchun 34. Cluster 6 included 5 genes, whose expression gradually decreased with grain development, and the expression level in Xinchun 34 was greater than that in Xinchun 26. Cluster 7 included 12 genes, and the expression levels of Cluster 7 gradually decreased with the development of grains on Lunar New Year 34; the expression levels were basically the same at 7 d and 14 d in New Spring 26, and the lowest expression was observed at 21 d. To further explore the relationship between these genes and gluten content, we calculated the correlation between gene expression and wheat glutenin and screened for absolute correlation coefficients greater than 0.5 for visualization (**Figure 6B**, **Supplementary Table S3**). A total of 25 genes were positively correlated with glutenin, with correlation coefficients ranging from 0.53 to 0.95, and 20 genes were negatively correlated with glutenin content, with correlation coefficients ranging from 0.50 to 0.95.

### 3.8 TF expression analysis

**Figure 7:**
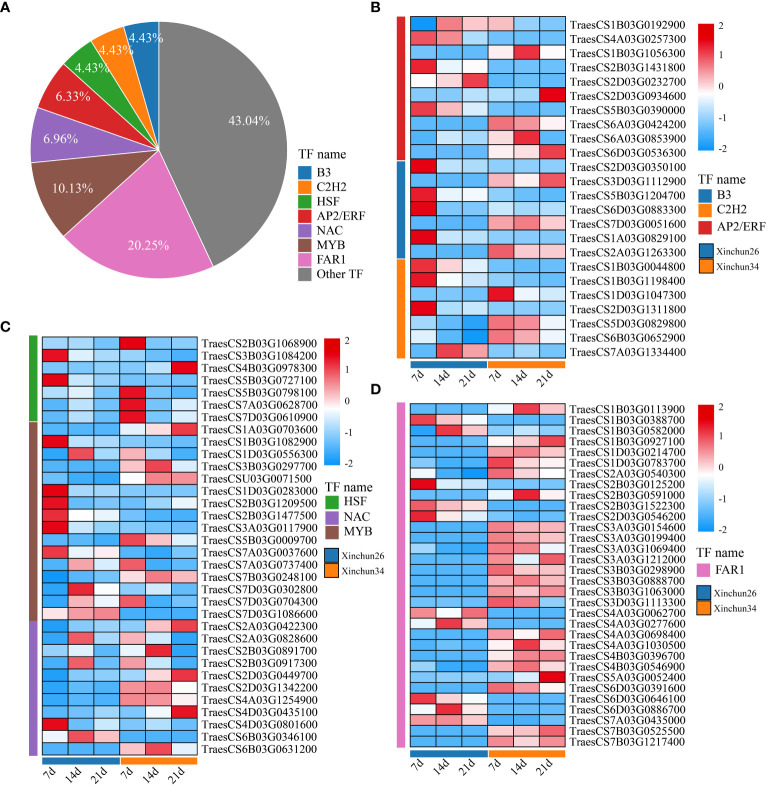
**(A)** The Xinchun 26 and Xinchun 34 difference TF pie charts are shown in **Figure 5B**. **Figure 5C** shows the expression heatmaps of B3, C2H2 and AP2/ERF between Xinchun 26 and Xinchun 34. Heatmap of HSF, NAC and MYB expression between Xinchun 26 and Xinchun 34, **Figure 5D**. FAR1 expression calorimetry between Xinchun 26 and Xinchun 34.

We analysed all the DEGs, which included B3 (4.43%), C2H2 (4.43%), AP2/ERF (6.33%), HSF (4.43%), NAC (6.96%), MYB (10.13%) and FAR1 (20.25%) (**Figure 7A**). The expression patterns of the differentially expressed TF genes are shown using a heatmap (**Figures 7B-D**). B3 exhibited completely opposite modes of expression in both materials (**Figure 7B**). C2H2 expression was downregulated in Xinchun 26 and upregulated or unchanged in Xinchun 34 (**Figure 6B**, **Supplementary Table S3**). The expression of most of the AP2/ERF genes gradually decreased with development (**Figure 6B**, **Supplementary Table S3**). The expression of all the genes in the HSF family except TraesCS4B03G0978300 was downregulated (**Figure 6C**, **Supplementary Table S3**). The expression pattern of NAC is complex, with expression downregulated in Xinchun 26, upregulated or unchanged in Xinchun 34, and upregulated in 26 (**Figure 6C**). MYB was expressed mainly in Yanghua plants after 14 and 21 d (**Figure 6C**). FAR1 was expressed mainly on Xinchun 34 (**Figure 6C**).

### 3.9 WGCNA

Based on the FPKM values of the genes, according to the soft threshold calculation results, β=7 was selected for network construction; a total of 12 coexpression modules were identified by combining and expressing similar modules via the dynamic shearing tree method, and each module is represented by a different colour (**Figure 7A**). Five of the 12 modules were strongly correlated with 7 d, 14 d and 21 d (**Figure 7B**), and four candidate genes were identified (*TraesCS7B03G1102000* (ATPase), *TraesCS1A03G0797600* (SpoU), *TraesCS2B03G0927000* (G6PD4) and *TraesCS4D03G0099800* (ADA1E)) (**Figure 7C**). Overall, 2 candidate genes were identified (*TraesCS5B03G1060800* (PUP4) and *TraesCS1B03G1120700* (PHO2)) (**Figure 7D**). Brown identified four candidate genes (*TraesCS1B03G0703000* (ERF), *TraesCS3D03G0849200* (PICALM4A), *TraesCS5B03G0681600* (O-glycosyl hydrolases) and *TraesCS3B03G0727200* (xanthine/uracil permease)) (**Figure 7E**). Four candidate genes were identified (*TraesCS6D03G0025600* (SWEET12), *TraesCS7B03G0941900* (DIR1-like), *TraesCS1A03G0577300* (CHX) and *TraesCS1A03G1007800* (DALL)) (**Figure 7F**). Four candidate genes were identified (*TraesCS1A03G0201700* (PIF3), *TraesCS2A03G1077700* (PPR), *TraesCS5B03G0905200* (COG) and *TraesCS3D03G0349800* (GRF)) (**Figure 7G**).

### 3.10 qRT−PCR of candidate genes

**Figure 8:**
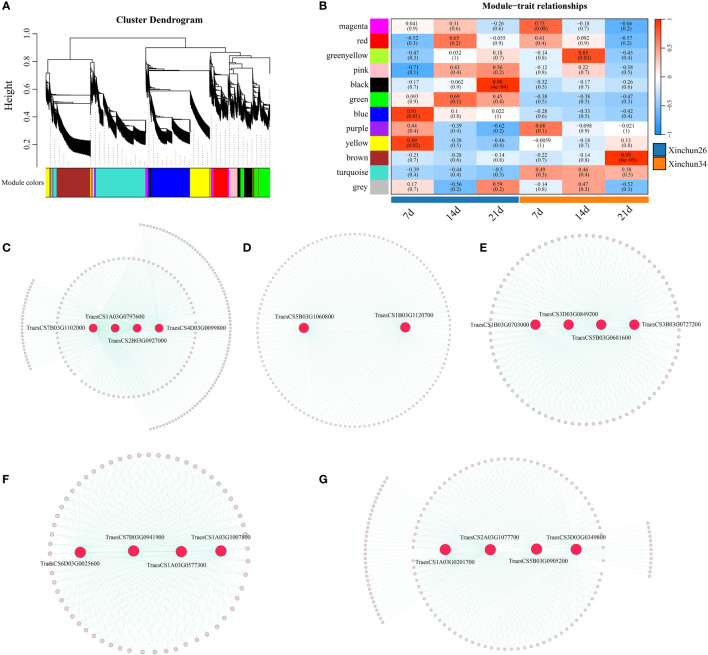
**(A)** A hierarchical clustering tree of genes was constructed based on the coexpression network analysis, **(B)** A heatmap of the correlations and significance between different developmental time points and materials, **(C)** The gene coexpression networks within the black modules, **(D)** The gene coexpression networks are shown within blue modules, **(E)** Gene coexpression networks within brown modules. **(F)** The gene coexpression networks within the green−yellow modules, **(G)** Gene coexpression networks within the green−yellow module.

Based on the 18 candidate genes associated with the gluten content of wheat grains mined by WGCNA, we performed qRT−PCR on Xinchun 26 and Xinchun 34, and 8 genes (*TraesCS7B03G1102000* (ATPase)*, TraesCS1A03G0797600* (SpoU)*, TraesCS2B03G0927000* (G6PD4)*, TraesCS5B03G1060800* (PUP4)*, TraesCS1B03G0703000* (ERF)*, TraesCS6D03G0025600* (SWEET12)*, TraesCS1A03G0201700* (PIF3) and *TraesCS1B03G1120700* (PHO2)) were significantly differentially expressed between the two materials (**Figure 8**, **Supplementary Figure S3**). The expression of *TraesCS7B03G1102000* (ATPase) and *TraesCS1B03G0703000* (ERF) decreased in both materials, and the expression in Xinchun 26 was significantly greater than that in Xinchun 34. Similarly, the expression of *TraesCS1A03G0797600* (SpoU) decreased in Xinchun 26 but did not change significantly in Xinchun 34. In Xinchun 26, TraesCS2B03G0927000 (G6PD4) expression decreased on 14 d and was 3 fold change than on 7 d. In Xinchun 34, TraesCS2B03G0927000 expression increased on 14d and was 4 fold change than that on 7d. In Xinchun 34, TraesCS5B03G1060800 (PUP4) expression increased on 21d and was 1.5 fold change than that on 7d. In Xinchun 26, TraesCS5B03G1060800 expression decreased on 14d and was 2 fold change than that on 7d. In both materials, *TraesCS6D03G0025600* (SWEET12), *TraesCS1A03G0201700* (PIF3) and *TraesCS1B03G1120700* (PHO2) increased significantly (**Figure 8**).

## 4 Discussion

Wheat is an important food crop that provides energy and a variety of nutrients, such as protein and dietary fibre, for humans (Chawade et al., 2018). In China, wheat is the main food for northerners, and more than 85% of wheat is used to make bread, biscuits, noodles and other flour products (Chawade et al., 2018; Zheng et al., 2018; Hackenberg et al., 2019)[1-3]. High-gluten wheat is suitable for making bread, medium-gluten wheat is suitable for making steamed buns and noodles, and low-gluten wheat is suitable for making biscuits (Chawade et al., 2018)[1]. As people pursue a higher quality of life, the demand for better quality specialty wheat is continually increasing (Zheng et al., 2018; Hackenberg et al., 2019). Therefore, an in-depth study of the quality formation mechanism of different types of wheat is highly important for the selection and breeding of wheat cultivars with high gluten levels. Wheat grain protein can be divided into nongluten protein (approximately 15%~20%) and gluten protein (approximately 80%~85%) (Zheng et al., 2018). The solubility of different reagents can be divided into four categories (i.e., albumin, globulin, gliadin and gluten) (Delcour et al., 2012; Zheng et al., 2018). Among these proteins, albumin and globulin are metabolic proteins that play a role in plant growth and seed development; moreover, both are structural proteins, the former giving rise to dough ductility and stickiness, and the latter giving rise to dough elasticity and strength (Delcour et al., 2012). Compared with those in the 7-d seed period after flowering, the expression of the four proteins at 14 d and 21 d increased significantly in both materials. The serum ALB concentration significantly differed among the three treatment groups, and the globulin concentration significantly differed between the two treatment groups at 21 d. The levels of gliadin and gluten were significantly greater in Xinchun 26 than in Xinchun 34 (**Figure 1**). These results showed that the gluten content of Xinchun 26 was significantly greater than that of Xinchun 34, and the contents of these four proteins also increased significantly with grain development.

**Figure 9:**
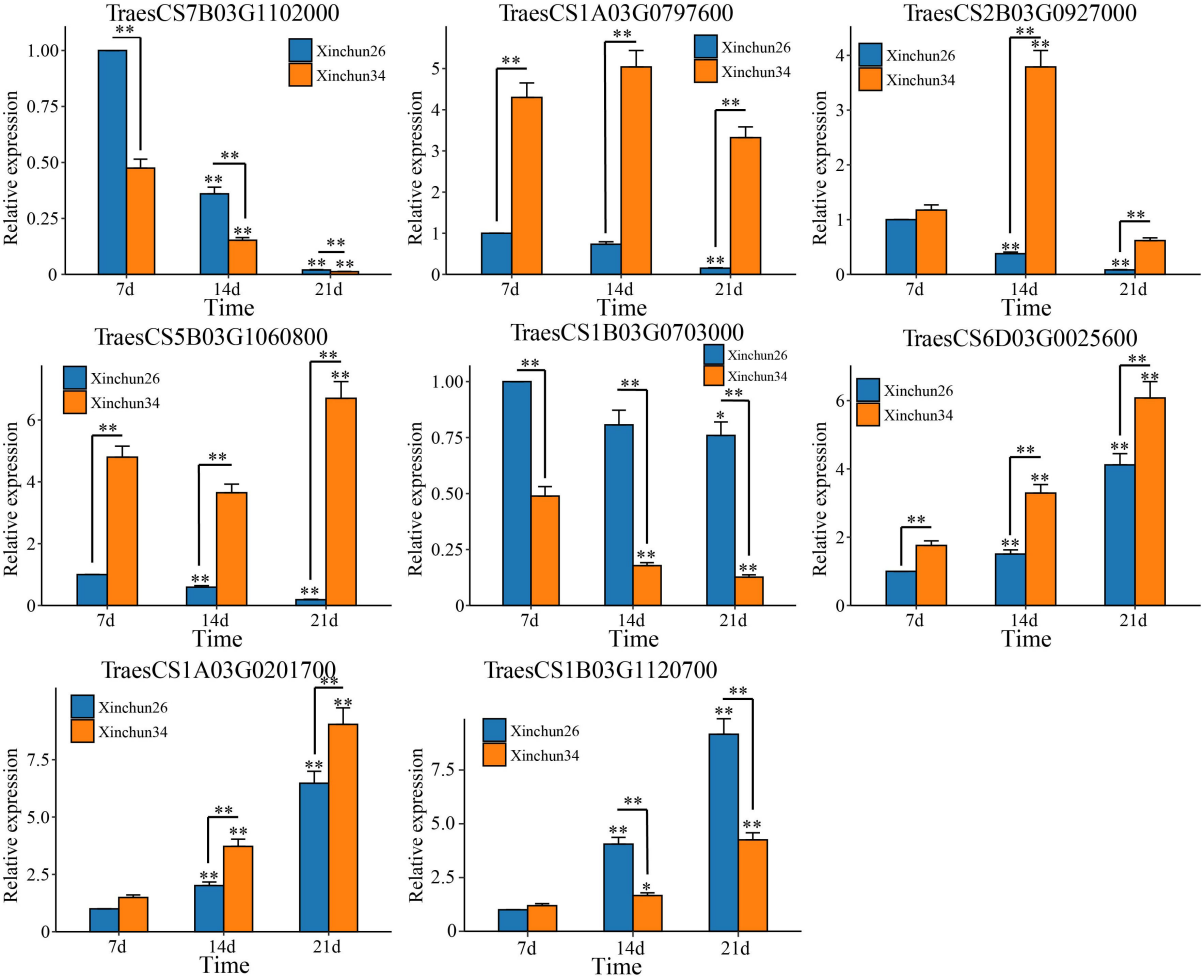
qRT−PCR of the wheat grain gluten content hub genes. The results are presented as the means ± SDs (n = 3, **P < 0.01, *P < 0.05).

To investigate the potential link between DEGs and metabolites during grain formation and gluten protein components in wheat, transcriptome and metabolome analyses were performed. PCA of the transcriptome and metabolome revealed that the differences between the periods were greater than the differences between materials, and the first principal component could distinguish between different materials and periods (**Supplementary Figure S1**, **Figure 3A**). There were 2697 DEGs in Xinchun 26 for the three periods, 1872 DEGs in Xinchun 34 for the three periods, and 5573 DEGs in the two materials for the three periods (**Figure 2**). According to the metabolomic differential analysis, 4 DRMs (3-methyl-L-histidine, L-arginine, L-citrulline, L-citrulline and L-asparagine) had the highest 7d content in Xinchun 26, which decreased with development (**Figure 4D**). The six DRMs (L-alanine, beta-alanine, 4-aminobutyric acid, xanthosine, N1-methyl-2-pyridone-5-carboxamide and N1-methyl-4-pyridone-3-carboxamide) had the highest 14d content in Xinchun 26 (**Figure 4D**). These genes and metabolites can provide a reference for the elucidation of gene and molecular mechanisms related to the content of gluten in wheat.

The accumulation of protein in wheat grains mainly depends on nitrogen metabolism and amino acid synthesis (Gao et al., 2021). In wheat, gluten is the main storage protein that determines gluten content (Yu et al., 2018). Gluten is first synthesized by the mRNA of the gluten gene family in the rough endoplasmic reticulum to form a 57 kDa precursor and then transported to the protein reservoir vacuole by Golgi modification, and the hydrolytic endonuclease in the vacuole results in the formation of an acidic subunit of 37-39 kDa and a basic subunit of 22-23 kDa (Yu et al., 2018; Gao et al., 2021). To this end, we identified 59 differentially expressed gluten synthesis-related genes, including 24 glutamine synthetases (GS), in wheat. Correlation analysis revealed that a total of 25 genes were positively correlated with glutenin, with correlation coefficients ranging from 0.53 to 0.95, and that 20 genes were negatively correlated with glutenin content, with correlation coefficients ranging from 0.50 to 0.95. GS is closely related to the high grain protein content and nitrogen efficiency of wheat, and its activity significantly affects protein and amino acid contents (Yin et al., 2022). The nitrogen absorbed by wheat is catalysed by GS to produce glutamine (Gln) by the ATP-dependent condensation reaction of ammonium with glutamic acid (Glu), which then provides the N group directly through Glu for the biosynthesis of proteins, amino acids, and other nitrogen-containing compounds (Nigro et al., 2016). We found that these DEGs related to gluten synthesis, especially GS, can be used as important candidate genes for improving wheat quality in the future.

Sugars are not only the energy source of plants but also important structural material components. Many kinds of sugars can also bind proteins to complex compounds (such as glycoproteins) and participate in cell recognition, intercellular material transport and other life activities, regulating plant growth and development (Kanwar and Jha, 2019; Nägele et al., 2022). The common enrichment pathways for DRMs and DEGs were starch and sucrose metabolism; the citrate cycle; carbon fixation in photosynthetic organisms; and alanine, aspartate and glutamate metabolism (**Figures 5A, B**). The sucrose content in Xinchun 34 was significantly greater than that in Xinchun 26, and the sucrose content in both materials at 14 d and 21 d was significantly greater than that in the seeds of Yanghua 7 d (**Figure 5D**). This means that the higher the sugar content of the wheat kernel is, the lower the gluten content. The sugar content of wheat not only reduces the yield of gluten but also may affect the composition and quality of gluten. Changes in light and dark times can significantly affect the synthesis of plant sugars because changes in light time can cause changes in the efficiency of plant cells to use light energy, which in turn affects cell division (Julius et al., 2017; Yoon et al., 2022). In addition, the photocycle can not only change the synthesis ability of photosynthetic pigments in plant cells but also affect the absorption and utilization of nutrients and other substances by cells and ultimately affect the synthesis and metabolism of the main active substances in cells (Ahmad, 2016; Hart et al., 2019). Our joint analysis also revealed that carbon fixation in photosynthetic organisms is an important pathway affecting the gluten content of wheat grains. The citrate cycle is a common metabolic pathway for the complete oxidation of the three main types of organic matter in the body: sugar, fat and protein (Fernie et al., 2004). The citrate cycle is a catabolic pathway that provides precursor molecules for the biosynthesis of several substances (Zhang and Fernie, 2018). For example, sugar and glycerol are metabolized in the body to produce α-ketoglutaric acid, oxaloacetic acid and other intermediate products of the tricarboxylic acid cycle; oxaloacetic acid is a precursor for the synthesis of aspartic acid; and α-ketoglutaric acid is a precursor for the synthesis of glutamic acid (Zhang and Fernie, 2023). Some amino acids can also produce sugars through different pathways through gluconeogenesis (Zhang and Fernie, 2023). These findings suggested that changes in the sugar and gluten contents of wheat grains may have been transformed by this process.

The method of combining transcriptome data with the WGCNA algorithm to study core genes related to plant growth and development and resistance has been widely used in the study of morphological formation and developmental regulatory mechanisms of plants, flowers, leaves and fruits, as well as the prediction of unknown gene functions (Dai et al., 2021; Wang et al., 2022; Yao et al., 2023). In this study, RNA-seq data from 18 Xinchun 26 and Xinchun 34 samples were used to screen 8 candidate genes related to the gluten content of wheat grains by combining wheat grains with the WGCNA algorithm combined with qRT−PCR. Among them, the expression of *TraesCS2B03G0927000* and *TraesCS5B03G1060800* in the low-gluten-content Xinchun 34 variety increased with grain development, while the expression in the high-gluten-content Xinchun 26 gradually decreased. These two genes may be the most important candidates for wheat gluten content. Based on the annotation of homologous Arabidopsis genes, we found that *TraesCS2B03G0927000* encodes a BTB/POZ domain-containing protein that mediates nutrient uptake and growth and development in Arabidopsis (Mao et al., 2017). *TraesCS5B03G1060800* encodes a purine permease that is involved in ATP-dependent cytokinin translocations and controls the spatiotemporal pattern of cytokinin signalling (Qi and Xiong, 2013). The depletion of ligands in the ectoplast leads to the inhibition of the cytokinin response (Qi and Xiong, 2013). Cytokinins can stimulate plant growth and flowering, control growth and differentiation, and delay ageing and are related to increased yield (Gupta and Rashotte, 2012). These two genes may regulate wheat grain gluten content through different mechanisms; however, the biological function of these genes (specifically, *TraesCS2B03G0927000* and *TraesCS5B03G1060800*) in regulating wheat grain gluten content requires further study. In conclusion, these findings provide new insights and ideas for the study of wheat grain gluten content and lay a foundation for in-depth analysis of the molecular mechanism of wheat grain gluten content.

## 5 Conclusion

In this study, the RNA-seq and metabolome of Xinchun 26 plants with high gluten content and low gluten content Xinchun 34 were analysed at 7, 14 and 21 d after flowering. Transcriptomic analysis revealed 5573 DEGs between the materials in the three periods, which were divided into two categories according to their expression patterns. Metabolomic analysis revealed that lipids, lipid-like molecules, phenylpropanoids and polyketides were the two most abundant metabolites, and the difference analysis revealed a total of 10 DRMs over the three periods. Combined RNA-seq and metabolome analysis revealed that starch and sucrose metabolism; the citrate cycle; carbon fixation in photosynthetic organisms; and alanine, aspartate and glutamate metabolism pathways were more important for determining the gluten content of wheat grains. By constructing a coexpression network, five specific modules that were strongly correlated with wheat grain development were identified, and eight candidate genes were screened via qRT−PCR. These findings provide new insights into the gluten content during wheat grain formation and reveal potential regulatory pathways and candidate genes involved in this developmental process.

## Data availability statement

The generated raw reads have been uploaded to NCBI’s SRA database and are available under the accession number PRJNA1021180. The datasets presented in this study can be found in online repositories. The names of the repository/repositories and accession number(s) can be found in the article/**Supplementary Material**.

## Author contributions

JS: Conceptualization, Data curation, Formal analysis, Investigation, Methodology, Software, Validation, Writing – original draft, Writing – review & editing. LW: Formal analysis, Investigation, Methodology, Writing – review & editing. ZhoW: Data curation, Formal analysis, Methodology, Writing – review & editing. JL: Formal analysis, Methodology, Writing – review & editing. HZ: Formal analysis, Methodology, Writing – review & editing. XG: Formal analysis, Methodology, Writing – review & editing. CW: Formal analysis, Methodology, Writing – review & editing. JX: Formal analysis, Methodology, Writing – review & editing. ZZ: Formal analysis, Methodology, Writing – review & editing. ZheW: Formal analysis, Methodology, Writing – review & editing. ZY: Formal analysis, Methodology, Writing – review & editing. ZX: Formal analysis, Methodology, Writing – review & editing. YZ: Conceptualization, Data curation, Formal analysis, Investigation, Methodology, Writing – review & editing. ZF: Conceptualization, Data curation, Formal analysis, Investigation, Methodology, Writing – review & editing.

## Supplementary material

Supplementary Figure 1 RNA-seq sample correlation and PCA.

Supplementary Figure 2 Scatter plot of the correlation between the gene expression levels from the transcriptome and qRT−PCR data.

Supplementary Figure 3 qRT−PCR analysis of the wheat grain gluten content hub genes; the results are presented as the means ± SDs (n = 3, **P < 0.01, *P < 0.05).

## References

[B1] Ahmad M. (2016). Photocycle and signaling mechanisms of plant cryptochromes. Curr. Opin. Plant Biol. 33, 108–115. doi: 10.1016/j.pbi.2016.06.013 27423124

[B2] Barker M. Rayens W. (2003). Partial least squares for discrimination. J. Chemometr 17, 166–173. doi: 10.1002/cem.785

[B3] Chawade A. Armoniené R. Berg G. Brazauskas G. Frostgård G. Geleta M.. (2018). A transnational and holistic breeding approach is needed for sustainable wheat production in the Baltic Sea region. Physiol. Plant 164, 442–451. doi: 10.1111/ppl.12726 29536550

[B4] Chen Q. F. Ya H. Y. Wang W. D. Jiao Z. (2014). RNA-seq reveals the downregulated proteins related to photosynthesis in growth-inhibited rice seedlings induced by low-energy N+ beam implantation. Genet. Mol. Res. 13, 7029–7036. doi: 10.4238/2014.March.26.9 24737518

[B5] Chen S. Zhou Y. Chen Y. Gu J. (2018). fastp: an ultra-fast all-in-one FASTQ preprocessor. Bioinformatics. 34, i884–i890. doi: 10.1093/bioinformatics/bty560 30423086 PMC6129281

[B6] Dai Y. Sun X. Wang C. Li F. Zhang S. Zhang H.. (2021). Gene co-expression network analysis reveals key pathways and hub genes in Chinese cabbage (*Brassica rapa* L.) during vernalization. BMC Genomics 22, 236. doi: 10.1186/s12864-021-07510-8 33823810 PMC8022416

[B7] Delcour J. A. Joye I. J. Pareyt B. Wilderjans E. Brijs K. Lagrain B. (2012). Wheat gluten functionality as a quality determinant in cereal-based food products. Annu. Rev. Food Sci. Technol. 3, 469–492. doi: 10.1146/annurev-food-022811-101303 22224557

[B8] Dunn W. B. Broadhurst D. Begley P. Zelena E. Francis-McIntyre S. Anderson N.. (2011). Procedures for large-scale metabolic profiling of serum and plasma using gas chromatography and liquid chromatography coupled to mass spectrometry. Nat. Protoc. 6, 1060–1083. doi: 10.1038/nprot.2011.335 21720319

[B9] Fernie A. R. Carrari F. Sweetlove L. J. (2004). Respiratory metabolism: glycolysis, the TCA cycle and mitochondrial electron transport. Curr. Opin. Plant Biol. 7, 254–261. doi: 10.1016/j.pbi.2004.03.007 15134745

[B10] Gao Y. An K. Guo W. Chen Y. Zhang R. Zhang X.. (2021). The endosperm-specific transcription factor TaNAC019 regulates glutenin and starch accumulation and its elite allele improves wheat grain quality. Plant Cell. 33, 603–622. doi: 10.1093/plcell/koaa040 33955492 PMC8136912

[B11] Gupta S. Rashotte A. M. (2012). Down-stream components of cytokinin signaling and the role of cytokinin throughout the plant. Plant Cell Rep. 31, 801–812. doi: 10.1007/s00299-012-1233-0 22315145

[B12] Hackenberg S. Vogel C. Scherf K. A. Jekle M. Becker T. (2019). Impact of altered starch functionality on wheat dough microstructure and its elongation behaviour. Food Chem. 290, 64–71. doi: 10.1016/j.foodchem.2019.03.016 31000057

[B13] Hart J. E. Sullivan S. Hermanowicz P. Petersen J. Diaz-Ramos L. A. Hoey D. J.. (2019). Engineering the phototropin photocycle improves photoreceptor performance and plant biomass production. Proc. Natl. Acad. Sci. U S A. 116, 12550–12557. doi: 10.1073/pnas.1902915116 31160455 PMC6589663

[B14] Hsu S. K. Tung C. W. (2017). RNA-seq analysis of diverse rice genotypes to identify the genes controlling coleoptile growth during submerged germination. Front. Plant Sci. 8. doi: 10.3389/fpls.2017.00762 PMC5430036 28555145

[B15] Huang Z. W. Yang Y. X. Huang L. H. Zhang S. Q. (2019). Pharmacokinetics and metabolism of icaritin in rats by UPLC-MS/MS. Food Sci. Nutr. 7, 4001–4006. doi: 10.1002/fsn3.1263 31890179 PMC6924312

[B16] Ji X. Jin B. Zhuang Z. Chang F. Wang F. Peng Y. (2022). Study on *zmRPN10* regulating leaf angle in maize by RNA-seq. Int. J. Mol. Sci. 24, 189. doi: 10.3390/ijms24010189 36613631 PMC9820655

[B17] Jiang Z. Zhang H. Jiao P. Wei X. Liu S. Guan S.. (2022). The integration of metabolomics and transcriptomics provides new insights for the identification of genes key to auxin synthesis at different growth stages of maize. Int. J. Mol. Sci. 23, 13195. doi: 10.3390/ijms232113195 36361983 PMC9659120

[B18] Jones W. P. Kinghorn A. D. (2012). Extraction of plant secondary metabolites. Methods Mol. Biol. 864, 341–366. doi: 10.1007/978-1-61779-624-1_13 22367903

[B19] Julius B. T. Leach K. A. Tran T. M. Mertz R. A. Braun D. M. (2017). Sugar transporters in plants: new insights and discoveries. Plant Cell Physiol. 58, 1442–1460. doi: 10.1093/pcp/pcx090 28922744

[B20] Kanehisa M. Goto S. (2000). KEGG: kyoto encyclopedia of genes and genomes. Nucleic Acids Res. 28, 27–30. doi: 10.1093/nar/28.1.27 10592173 PMC102409

[B21] Kanwar P. Jha G. (2019). Alterations in plant sugar metabolism: signatory of pathogen attack. Planta. 249, 305–318. doi: 10.1007/s00425-018-3018-3 30267150

[B22] Kusser W. Javorschi S. Gleeson M. A. (2006). Real-time RT-PCR: cDNA synthesis. Cold Spring Harbor Protoc 2006. pdb-prot4114. doi: 10.1101/pdb.prot4114 22485518

[B23] Li H. Li Y. Song L. Cheng J. Ge J. Yu X.. (2022). Effects of tebuconazole application at different growth stages on rice grain quality of rice-based untargeted metabolomics. Chemosphere. 303, 134920. doi: 10.1016/j.chemosphere.2022.134920 35588883

[B24] Liu S. Wang Z. Zhu R. Wang F. Cheng Y. Liu Y. (2021). Three differential expression analysis methods for RNA sequencing: limma, edgeR, DESeq2. J. Vis. Exp. 18, 175. doi: 10.3791/62528 34605806

[B25] Ma L. Zhang M. Chen J. Qing C. He S. Zou C.. (2021). GWAS and WGCNA uncover hub genes controlling salt tolerance in maize (*Zea mays* L.) seedlings. Theor. Appl. Genet. 134, 3305–3318. doi: 10.1007/s00122-021-03897-w 34218289

[B26] Mao H. Aryal B. Langenecker T. Hagmann J. Geisler M. Grebe M. (2017). Arabidopsis BTB/POZ protein-dependent PENETRATION3 trafficking and disease susceptibility. Nat. Plants. 3, 854–858. doi: 10.1038/s41477-017-0039-z 29085068

[B27] Nägele T. Gibon Y. Hir. R. (2022). Plant sugar metabolism, transport and signalling in challenging environments. Physiol. Plant 174, e13768. doi: 10.1111/ppl.13768 36281839

[B28] Nigro D. Fortunato S. Giove S. L. Paradiso A. Gu Y. Q. Blanco A.. (2016). Glutamine synthetase in durum wheat: genotypic variation and relationship with grain protein content. Front. Plant Sci. 7. doi: 10.3389/fpls.2016.00971 PMC4942471 27468287

[B29] Pankievicz V. C. Camilios-Neto D. Bonato P. Balsanelli E. Tadra-Sfeir M. Z. Faoro H.. (2016). RNA-seq transcriptional profiling of Herbaspirillum seropedicae colonizing wheat (*Triticum aestivum*) roots. Plant Mol. Biol. 90, 589–603. doi: 10.1007/s11103-016-0430-6 26801330

[B30] Peng L. Gao W. Song M. Li M. He D. Wang Z. (2022). Integrated Metabolome and Transcriptome Analysis of Fruit Flavor and Carotenoids Biosynthesis Differences Between Mature-Green and Tree-Ripe of cv. "Golden Phoenix" Mangoes (*Mangifera indica* L.). Front. Plant Sci. 13. doi: 10.3389/fpls.2022.816492 PMC8907839 35283889

[B31] Pertea M. Kim D. Pertea G. M. Leek J. T. Salzberg S. L. (2016). Transcript-level expression analysis of RNA-seq experiments with HISAT, StringTie and Ballgown. Nat. Protoc. 11, 1650–1667. doi: 10.1038/nprot.2016.095 27560171 PMC5032908

[B32] Prakash S. Kumar M. Radha. Kumar S. Jaconis S. Parameswari E.. (2023) The resilient cotton plant: uncovering the effects of stresses on secondary metabolomics and its underlying molecular mechanisms. Funct Integr Genomics. 23(2):183. doi: 10.1007/s10142-023-01118-9 37233833

[B33] Qi Z. Xiong L. (2013). Characterization of a purine permease family gene OsPUP7 involved in growth and development control in rice. J. Integr. Plant Biol. 55, 1119–1135. doi: 10.1111/jipb.12101 24034337

[B34] Shannon P. Markiel A. Ozier O. Baliga N. S. Wang J. T. Ramage D.. (2003). Cytoscape: a software environment for integrated models of biomolecular interaction networks. Genome Res. 13, 2498–2504. doi: 10.1101/gr.1239303 14597658 PMC403769

[B35] Stelpflug S. C. Sekhon R. S. Vaillancourt B. Hirsch C. N. Buell C. R. de Leon N.. (2016). An Expanded Maize Gene Expression Atlas based on RNA Sequencing and its Use to Explore Root Development. Plant Genome. 9 (1). doi: 10.3835/plantgenome2015.04.0025 27898762

[B36] Wan H. Qian J. Zhang H. Lu H. Li O. Li R.. (2022). Combined transcriptomics and metabolomics analysis reveals the molecular mechanism of salt tolerance of huayouza 62, an elite cultivar in rapeseed (*Brassica napus* L.). Int. J. Mol. Sci. 23, 1279. doi: 10.3390/ijms23031279 35163202 PMC8836002

[B37] Wang Y. Liu W. Wang X. Yang R. Wu Z. Wang H.. (2020). MiR156 regulates anthocyanin biosynthesis through *SPL* targets and other microRNAs in poplar. Hortic. Res. 7, 118. doi: 10.1038/s41438-020-00341-w 32821401 PMC7395715

[B38] Wang Z. Yang H. Ma Y. Jiang G. Mei X. Li X.. (2022). WGCNA analysis revealing molecular mechanism that bio-organic fertilizer improves pear fruit quality by increasing sucrose accumulation and reducing citric acid metabolism. Front. Plant Sci. 13. doi: 10.3389/fpls.2022.1039671 PMC9606799 36311108

[B39] Wen B. Mei Z. Zeng C. Liu S. (2017). metaX: a flexible and comprehensive software for processing metabolomics data. BMC Bioinf. 18, 183. doi: 10.1186/s12859-017-1579-y PMC5361702 28327092

[B40] Westerhuis J. A. Hoefsloot H. C. Smit S. Vis D. J. Smilde A. K. van Velzen E. J.. (2008). Assessment of PLSDA cross validation. Metabolomics 4, 81–89. doi: 10.1007/s11306-007-0099-6

[B41] Wu S. Wu D. Song J. Zhang Y. Tan Q. Yang T.. (2022). Metabolomic and transcriptomic analyses reveal new insights into the role of abscisic acid in modulating mango fruit ripening. Hortic. Res. 9, uhac102. doi: 10.1093/hr/uhac102 35795388 PMC9250656

[B42] Yao Y. Xiong E. Qu X. Li J. Liu H. Quan L.. (2023). WGCNA and transcriptome profiling reveal hub genes for key development stage seed size/oil content between wild and cultivated soybean. BMC Genomics 24, 494. doi: 10.1186/s12864-023-09617-6 37641045 PMC10463976

[B43] Yin H. Sun Q. Lu X. Zhang L. Yuan Y. Gong C.. (2022). Identification of the glutamine synthetase (GS) gene family in four wheat species and functional analysis of Ta4D.GSe in Arabidopsis thaliana. Plant Mol. Biol. 110, 93–106. doi: 10.1007/s11103-022-01287-4 35716232 PMC9468116

[B44] Yoon J. Cho L. H. Tun W. Jeon J. S. An G. (2012). Sucrose signaling in higher plants. Plant Sci. 302, 110703. doi: 10.1016/j.plantsci.2020.110703 33288016

[B45] Yu Z. Islam S. She M. Diepeveen D. Zhang Y. Tang G.. (2018). Wheat grain protein accumulation and polymerization mechanisms driven by nitrogen fertilization. Plant J. 96, 1160–1177. doi: 10.1111/tpj.14096 30230644

[B46] Yu X. Liu H. Xu X. Hu Y. Wang X. Wen C. (2021). Pharmacokinetics of yunaconitine and indaconitine in mouse blood by UPLC-MS/MS. J. Chromatogr B Analyt Technol. BioMed. Life Sci. 1179, 122840. doi: 10.1016/j.jchromb.2021.122840 34225245

[B47] Zhang Y. Fernie A. R. (2018). On the role of the tricarboxylic acid cycle in plant productivity. J. Integr. Plant Biol. 60, 1199–1216. doi: 10.1111/jipb.12690 29917310

[B48] Zhang Y. Fernie A. R. (2023). The role of TCA cycle enzymes in plants. Adv. Biol. (Weinh). 7, e2200238. doi: 10.1002/adbi.202200238 37341441

[B49] Zhao L. Zhang B. Huang H. Huang W. Zhang Z. Wang Q.. (2022). Metabolomic and transcriptomic analyses provide insights into metabolic networks during cashew fruit development and ripening. Food Chem. 404, 134765. doi: 10.1016/j.foodchem.2022.134765 36444096

[B50] Zheng T. Qi P. F. Cao Y. L. Han Y. N. Ma H. L. Guo Z. R.. (2018). Mechanisms of wheat (*Triticum aestivum*) grain storage proteins in response to nitrogen application and its impacts on processing quality. Sci. Rep. 8, 11928. doi: 10.1038/s41598-018-30451-4 30093727 PMC6085318

