# Supplementary figures and images for: Comparison of the transcriptome and metabolome of wheat (Triticum aestivum L.) proteins content during grain formation provides insight
Source: Front Plant Sci. 2024 Jan 18;14:1309678. doi: 10.3389/fpls.2023.1309678 (PMC10830700; doi:10.3389/fpls.2023.1309678)

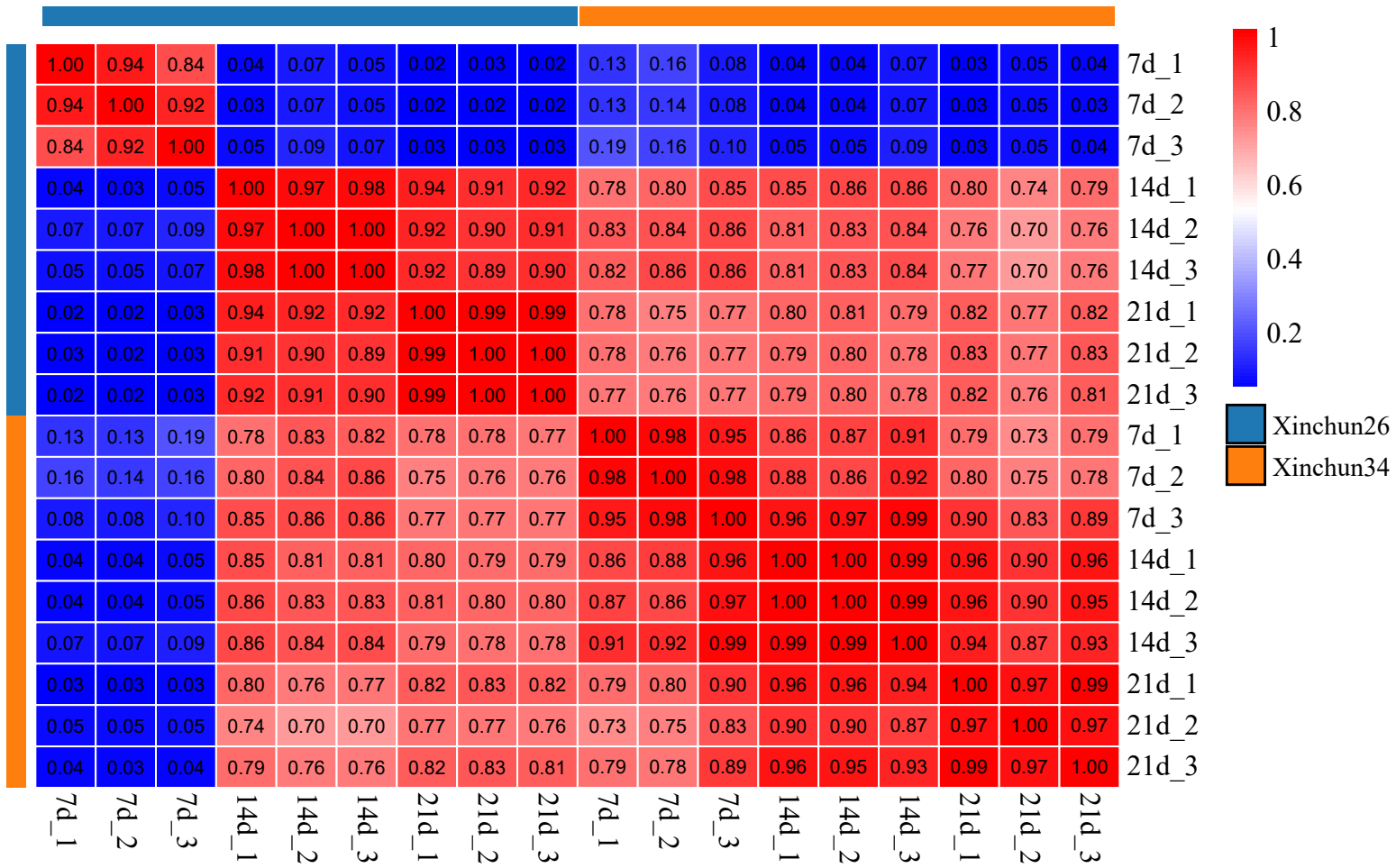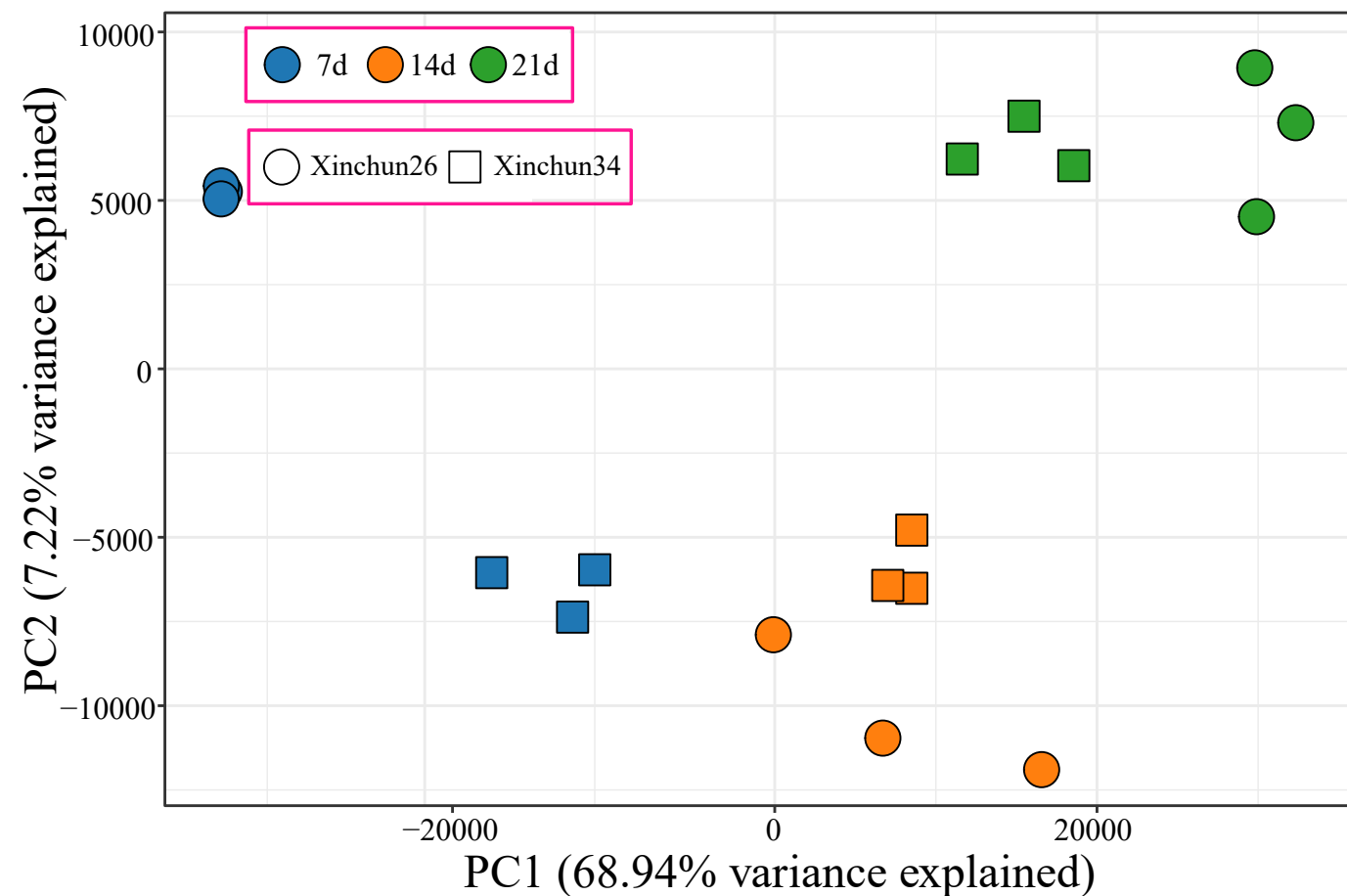

Supplement: Supplementary Figure 1 — RNA-seq sample correlation and PCA. [file DataSheet_1.pdf]

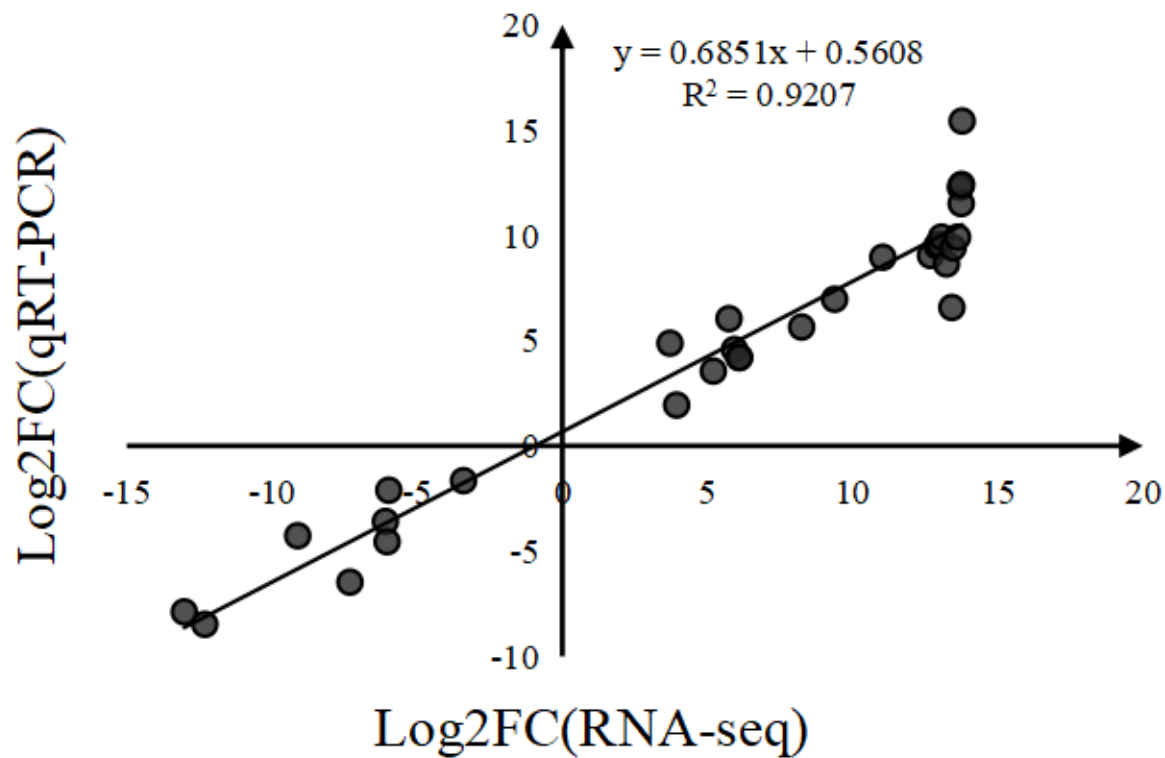

Supplement: Supplementary Figure 2 — Scatter plot of the correlation between the gene expression levels from the transcriptome and qRT−PCR data. [file DataSheet_2.pdf]
