# Supplementary material for: Comparison of the transcriptome and metabolome of wheat (Triticum aestivum L.) proteins content during grain formation provides insight
Source: Front Plant Sci. 2024 Jan 18;14:1309678. doi: 10.3389/fpls.2023.1309678 (PMC10830700; doi:10.3389/fpls.2023.1309678)
Supplement: Supplementary Figure 3 — qRT−PCR analysis of the wheat grain gluten content hub genes; the results are presented as the means ± SDs (n = 3, **P < 0.01, *P < 0.05). [file DataSheet_3.pdf]

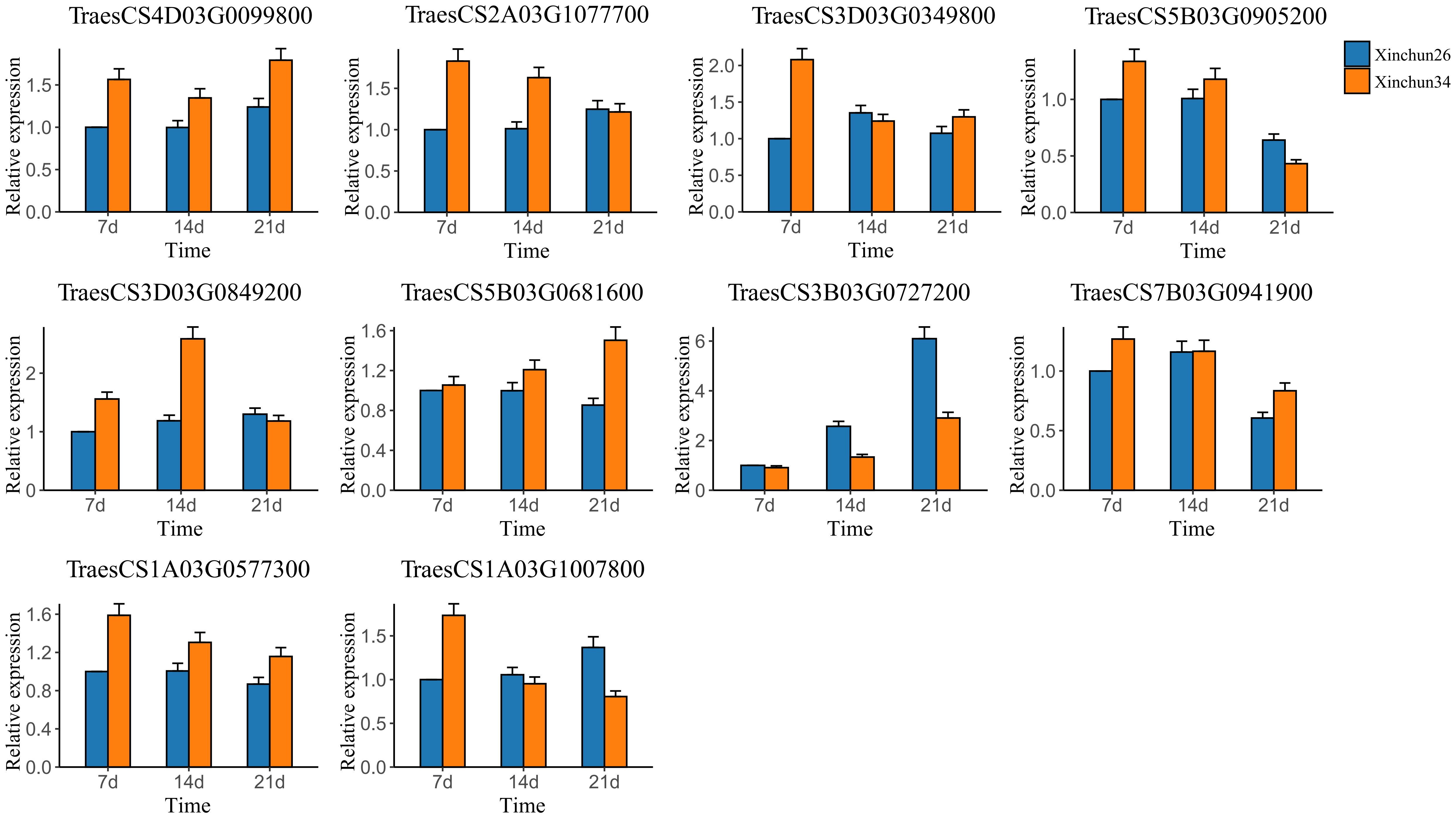
TraesCS4D03G0099800
TraesCS2A03G1077700
TraesCS3D03G0349800
TraesCS5B03G0905200
TraesCS3D03G0849200
TraesCS5B03G0681600
TraesCS3B03G0727200
TraesCS7B03G0941900
TraesCS1A03G0577300
TraesCS1A03G1007800
Relative expression
Time
7d
14d
21d
Xinchun26
Xinchun34
